# A multi-modal fusion model with enhanced feature representation for chronic kidney disease progression prediction

**DOI:** 10.1093/bib/bbaf003

**Published:** 2025-02-06

**Authors:** Yixuan Qiao, Hong Zhou, Yang Liu, Ruixuan Chen, Xiaodong Zhang, Sheng Nie, Fan Fan Hou, Yi Zhao, Xin Xu, Lianhe Zhao

**Affiliations:** Institute of Computing Technology, Chinese Academy of Sciences (ICT), 6 Kexueyuan Nanlu, Zhongguancun, Haidian, Beijing 100190, China; University of Chinese Academy of Sciences, No. 1 Yanqihu East Rd, Huairou District, Beijing 101408, PR China; National Clinical Research Center for Kidney Disease, State Key Laboratory of Organ Failure Research, Nanfang Hospital, Southern Medical University, No. 1838 Guangzhou North Avenue, Baiyun District, Guangzhou 510515, China; Institute of Computing Technology, Chinese Academy of Sciences (ICT), 6 Kexueyuan Nanlu, Zhongguancun, Haidian, Beijing 100190, China; University of Chinese Academy of Sciences, No. 1 Yanqihu East Rd, Huairou District, Beijing 101408, PR China; National Clinical Research Center for Kidney Disease, State Key Laboratory of Organ Failure Research, Nanfang Hospital, Southern Medical University, No. 1838 Guangzhou North Avenue, Baiyun District, Guangzhou 510515, China; National Clinical Research Center for Kidney Disease, State Key Laboratory of Organ Failure Research, Nanfang Hospital, Southern Medical University, No. 1838 Guangzhou North Avenue, Baiyun District, Guangzhou 510515, China; National Clinical Research Center for Kidney Disease, State Key Laboratory of Organ Failure Research, Nanfang Hospital, Southern Medical University, No. 1838 Guangzhou North Avenue, Baiyun District, Guangzhou 510515, China; National Clinical Research Center for Kidney Disease, State Key Laboratory of Organ Failure Research, Nanfang Hospital, Southern Medical University, No. 1838 Guangzhou North Avenue, Baiyun District, Guangzhou 510515, China; Institute of Computing Technology, Chinese Academy of Sciences (ICT), 6 Kexueyuan Nanlu, Zhongguancun, Haidian, Beijing 100190, China; University of Chinese Academy of Sciences, No. 1 Yanqihu East Rd, Huairou District, Beijing 101408, PR China; National Clinical Research Center for Kidney Disease, State Key Laboratory of Organ Failure Research, Nanfang Hospital, Southern Medical University, No. 1838 Guangzhou North Avenue, Baiyun District, Guangzhou 510515, China; Institute of Computing Technology, Chinese Academy of Sciences (ICT), 6 Kexueyuan Nanlu, Zhongguancun, Haidian, Beijing 100190, China; University of Chinese Academy of Sciences, No. 1 Yanqihu East Rd, Huairou District, Beijing 101408, PR China

**Keywords:** multi-modal, deep learning, chronic kidney disease, progression prediction, multi-omics, computational pathology

## Abstract

Artificial intelligence (AI)-based multi-modal fusion algorithms are pivotal in emulating clinical practice by integrating data from diverse sources. However, most of the existing multi-modal models focus on designing new modal fusion methods, ignoring critical role of feature representation. Enhancing feature representativeness can address the noise caused by modal heterogeneity at the source, enabling high performance even with small datasets and simple architectures. Here, we introduce DeepOmix-FLEX (Fusion with Learning Enhanced feature representation for X-modal or FLEX in short), a multi-modal fusion model that integrates clinical data, proteomic data, metabolomic data, and pathology images across different scales and modalities, with a focus on advanced feature learning and representation. FLEX contains a Feature Encoding Trainer structure that can train feature encoding, thus achieving fusion of inter-feature and inter-modal. FLEX achieves a mean AUC of 0.887 for prediction of chronic kidney disease progression on an internal dataset, exceeding the mean AUC of 0.727 using conventional clinical variables. Following external validation and interpretability analyses, our model demonstrated favorable generalizability and validity, as well as the ability to exploit markers. In summary, FLEX highlights the potential of AI algorithms to integrate multi-modal data and optimize the allocation of healthcare resources through accurate prediction.

## Introduction

Over the past 30 years, chronic kidney disease (CKD) as a cause of death has increased and contributes 1.35% of the global burden of disability-adjusted life years lost [1]. Within the context of an aging population, CKD prevalence is increasing and involves 10% of the global population [2–4].

Clinical treatment decisions for CKD are challenging due to the multiple pathologic types and complexity of the patient profile. Many models have been developed to predict CKD progression [5], most using data recorded in electronic medical records (EMR). Kidney Failure Risk Equation (KFRE) selected four clinical and biochemical variables to predict the risk of kidney failure [6]. Tangri et al. fused demographic, clinical, and laboratory data to improve prediction accuracy [7]. Several studies have leveraged machine learning algorithms (e.g. random forests, logistic regression, support vector machines) to improve feature complexity and expand the range of variables that can be analyzed [8–12]. Besides, some studies have used several data modalities other than clinical data. Bienaimé et al. used urine biomarkers to assess CKD progression [13]. Agarwal et al. examined protein biomarkers in blood and urine samples [14]. Looker et al. integrated serum biomarkers and clinical variables to predict rapid progression of CKD in patients with type 2 diabetes [15]. Kondo et al. analyzed spatial proteomics and found that protein expression, cellular phenotypic composition, and microenvironmental structure changed with the progression of diabetic nephropathy [16]. However, in clinical practice, doctors need to integrate a variety of information, such as disease descriptions, examination reports, and pathology images, in order to make decisions. Existing models, which analyze and utilize each modality data independently, do not pay attention to the complementary information between modalities, and the potential of multi-modal data in analyzing the disease progression of patients with CKD has not been realized.

Artificial intelligence (AI) algorithms have been shown to effectively analyze big data for diagnosing and treating diseases [17–19]. In particular, AI-based multi-modal algorithms can discover the internal patterns between different modal data within a short training time, giving solutions to population health problems that were previously thought to be impossible, e.g. Alzheimer’s disease, and cardiovascular disease [20–22]. Vanguri et al. collected patient-level features from histology, radiology, genomics and biomarkers using both manual and machine learning methods and fed the extracted features together into a modal fusion model [23]. Boehm et al. improved risk stratification of high-grade serous ovarian cancer using radiological features extracted by traditional image methods, annotation of histological images, and binary encoding of clinical data [24]. Chen et al. integrated histology-genomics analysis with patch-level image features and raw molecular features on pan-cancer [25]. But these methods only address the problem of how to fuse the data from each modality together. The representation of the features is fixed before they are fed into the model’s fusion module. Suitable data fusion methods can improve the expressive ability of the model, but if there is a lot of noise in the input features also cannot make effective prediction. Zhou et al. have considered fusion at the feature level, but they need to build the model on a large dataset [17]. There is an objective difficulty in that as the data becomes more modal, the number of samples that can match multiple modalities for use is declining. Therefore, it is essential to explore methods for enhancing feature representativeness while integrating additional modalities, particularly when working with small datasets.

**Figure 1:**
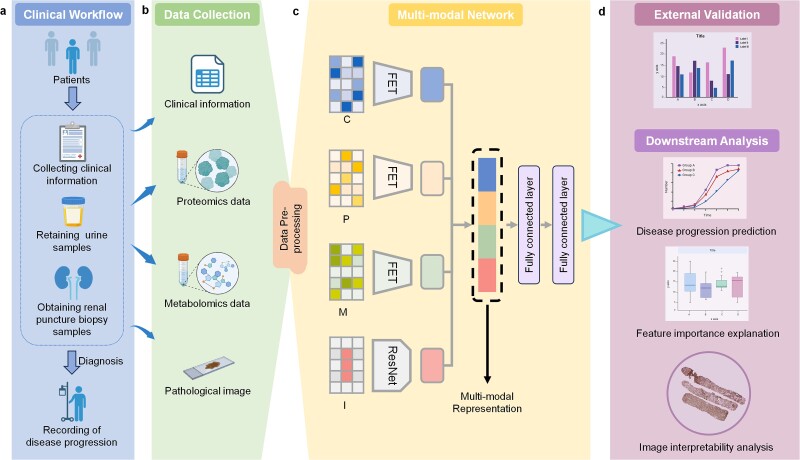
Overview of the FLEX. (a) The multimodal data are derived from EMR, urine samples and kidney biopsy samples that are retained by patients during medical procedures. Patients diagnosed with chronic kidney disease will receive regular follow-up to document disease progression over a three-year period. (b) Four modalities of data were extracted from records and samples. Clinical data from EMR, proteomics and metabolomics data from urine samples, and PASM-stained digital pathology images from kidney samples, respectively. (c) The FLEX receives the preprocessed data of the four modalities, uses an end-to-end training approach to extract the representative vectors for four modalities separately, and fuses the vectors to generate a multimodal representation to be fed into a classifier stacked with two fully-connected layers to predict the three-year progression of a patient’s CKD. (d) FLEX can perform the downstream tasks of progression prediction, modal importance analysis, feature importance interpretation, and image visualization. (C: Clinical data, P: Proteomics data, M: Metabolomics data, I: Pathology images).

In summary, we have found that in the field of medical multi-modal algorithms, high heterogeneity among modalities and instability in the quantity and quality of data are the key reasons limiting model performance. There is a lack of an algorithm that can quickly and efficiently fuse multi-modal data directly on small datasets without complex data preprocessing. In CKD applications, the types of data currently used are few. So, in this paper, we introduce FLEX, a deep learning-based multi-modal data fusion model designed to predict CKD progression. FLEX uniquely integrates pathology images, clinical data, proteomics data, and metabolomics data through advanced fusion learning to create a comprehensive representation of disease progression. The X in FLEX means that FLEX can be flexibly applied in the case of 1 or 2 or 3 or 4 modal data. FLEX innovates the encoding of features to improve the efficiency of feature fusion, making the model optimize both the model structure and feature encoding during the training process, thus enhancing feature representativeness. To the best of our knowledge, FLEX is the first end-to-end multi-modal analytical model in the medical field that simultaneously learns from multiple data sources with enhanced feature representativeness, as illustrated in Fig. 1. We validate the effectiveness of the model through multiple comparisons and ablation experiments. Additionally, we assess the model’s generalizability on an external dataset, confirming its robustness and reliability. We also visualize the modal and feature importance distributions, which are crucial for interpretable analysis of multi-modal models. In summary, our study highlights the significant advantages of using AI to integrate multi-modal data, showcasing FLEX’s ability to accurately predict CKD progression and advance personalized medicine approaches.

## Results

### Overview

To address the challenge of integrating multi-source, multi-scale data, we developed FLEX, a pioneering multi-modal interpretable deep learning model for predicting and interpreting CKD progression. FLEX uniquely integrates clinical data (C), proteomic data (P), metabolomic data (M), and digital pathology images (I) to forecast CKD progression over 3 years, as illustrated in Fig. 1b. FLEX introduces a feature encoding trainer (FET) specifically developed for tabular form data. FET allows FLEX to simultaneously train and update the encoding of features while training the model parameters end-to-end, thus enhancing the extraction of high-dimensional abstract features (Fig. 1c). We trained our model on the internal dataset using five-fold cross-validation and reported six performance metrics. To validate the model, we performed ablation analysis with different feature numbers, modal combinations, modal fusion methods, and base models. In particular, we collected an external dataset containing only tabular data to validate model robustness. Upon completing the model construction, we visualized and interpreted the importance of features affecting progress across modalities. We also compared the differences in feature importance across populations using feature aggregation and gradient-based methods (Fig. 1d). FLEX represents a significant advancement in the CKD field, offering both high clinical relevance and computational efficacy as one of the first end-to-end multi-modal models to integrate diverse data types for improved disease prediction and understanding.

### Dataset construction

We collected 259 CKD patients from May 2013 through November 2021 at Nanfang Hospital as the internal dataset. CKD progression was considered a binary endpoint, and patients were divided into progression and non-progression groups based on their follow-up records according to their CKD progression over three years (‘Progression definition’ section in Supplementary Data). The progression group consisted of 106 patients, while the non-progression group consisted of 153 patients. Each patient had at least one electronic medical record, one urine sample and one kidney biopsy sample (Fig. 1a). For duplicate data, keeping records belonging to the progression group or those with an earlier sample acquisition time was prioritized. The internal dataset collected data in four modalities: clinical data, proteomics data, metabolomics data, and image data, which is the most informative CKD progression study data. The EMR provided clinical data, including demographic information, past medical history, medication history, laboratory test results, and pathologic diagnosis. Urine samples were used for proteomic sequencing and metabolic analysis to generate proteomics and metabolomics data, respectively. Kidney biopsy samples were used to obtain digital pathology images with 20× or 40× Periodic Acid-silver Methenamine (PASM) staining. The data processing pipeline is shown in Supplementary Fig. S1 and ‘Dataset description’ section in Supplementary Data.

The external dataset contained 81 patients at Nanfang Hospital from June 2013 through January 2022, with a similar distribution of CKD progression categories as the internal dataset. Three modalities of tabular data were included in the external dataset: clinical data, proteomics data, and metabolomics data, using the same processing methods as in the internal dataset. The characteristics of on the internal dataset and external dataset are summarized in Supplementary Table S1. There is no intersection between the internal dataset and the external dataset, as well as, between the training, test and validation sets of the internal dataset.

### Multi-modal fusion improves the performance of CKD progression prediction

**Figure 2:**
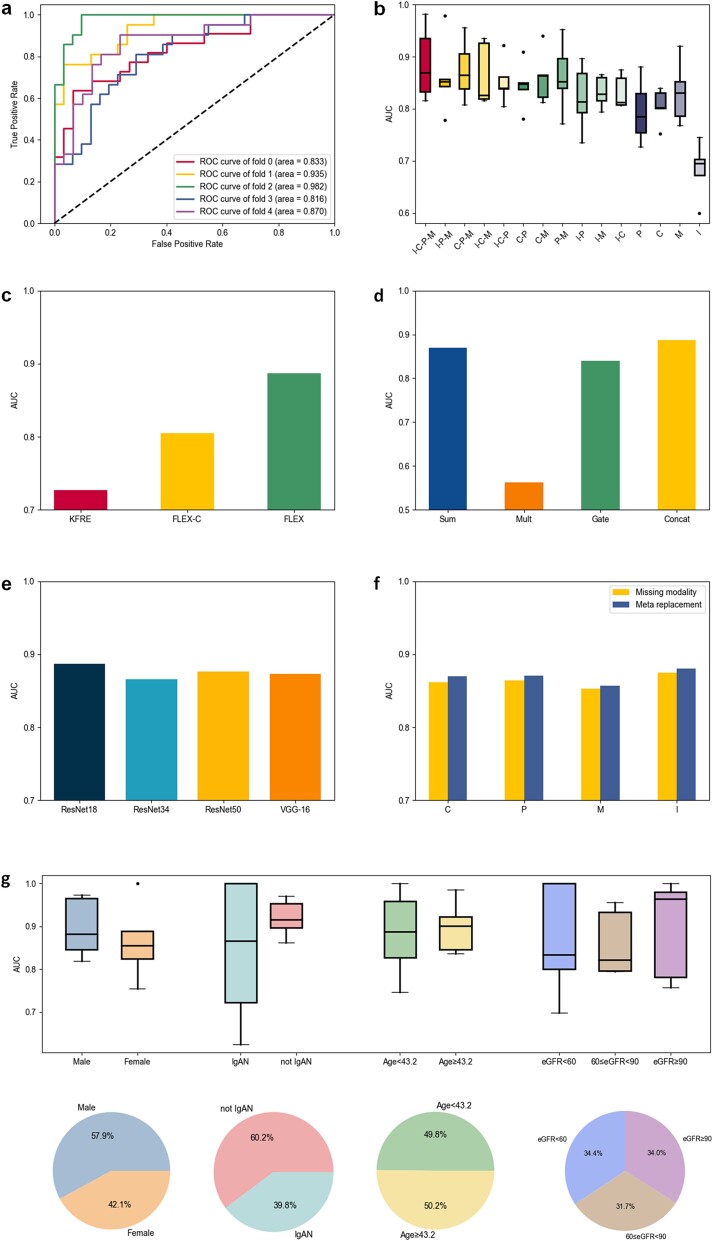
Performance of the FLEX on internal dataset. (a) Receiver operating characteristic (ROC) plots for FLEX with five-fold cross-validation based on four-modal data for predicting three-year disease progression in CKD patients. (b) AUC performance distribution of different modal combinations in five-fold cross-validation for FLEX. (c) Comparison of KFRE, FLEX-C (FLEX with only clinical modality inputs) and FLEX performance using mean AUC. (d) Performance comparison of FLEX using different modal fusion methods. (e) Performance comparison of FLEX using different base image models. (f) Comparison of mean AUC performance between missing individual modal data and replacing the missing modality with meta module for both cases. (g) Performance comparisons and percentage statistics using gender, if IgAN, age and eGFR to categorize patients into different groups.

We first evaluated the performance of FLEX on the internal dataset using five-fold cross-validation. FLEX achieved a mean area under the receiver operating characteristic (AUC) of 0.887 when integrating data across the four modalities C, P, M, and I (Fig. 2a). To further explore the impact of different data modality combinations, we maintained the model architecture and data splitting while performing experiments with various modality combinations. The AUC distributions for these experiments are shown in Fig. 2b (see Supplementary Table S2 for full performance demonstration). Four-modality fusion(I-C-P-M)‘s mean AUC exceeded unimodal I, C, P, and M by, respectively, 0.204, 0.082, 0.092, and 0.056. We observed that the model’s performance improves as more data modalities are integrated. Specifically, in the comparison of mean AUC with single-modal performance as the baseline, we find that the image model obtained the most improvement by superimposing other modal data (i.e. C, P, M, C-P, C-M, P-M, C-P-M). We attribute this improvement to the substantial differences in input forms and feature extraction methods between image data and other modalities, as well as the enhancement provided by tabular data in the model’s ability to interpret images. In contrast, when we added one modality’s data to any combination of the other modality’s data, we found that adding proteomics data provided the greatest performance improvement, possibly because proteomics features are more independent of the other modalities, which creates feature complementarities and leads performance improvements. Overall, clinical data, image data, proteomics, and metabolomics data, represent individual, tissue, and molecular scale features, respectively. Despite the high heterogeneity in data structure and content across these modalities, FLEX can integrate multimodal data and investigates underlying associations to enhance the accuracy of predicting CKD progression.

In order to further explore the performance of FLEX under different subgroups, we have statistically analyzed the results of the best trained models on the test set according to gender (male, female), pathological diagnosis (IgA nephropathy [IgAN], not IgAN), age (grouped by median), and estimated glomerular filtration rate (eGFR) grading, respectively. The mean AUC values were as follows: male 0.897 versus female 0.865, younger 0.884 versus older 0.898, IgAN 0.843 versus non-IgAN 0.920, and low eGFR 0.866 versus median eGFR 0.860 versus high eGFR 0.897 (Fig. 2g). No significant differences were observed within any subgroup, highlighting the robustness of our model.

We compared FLEX, FLEX using only clinical data (FLEX-C), and the clinically common KFRE model [6]. FLEX and FLEX-C had mean AUCs that improved compared to KFRE by 0.160 and 0.082 (Fig. 2c). Among age, sex, urinary albumin to creatinine ratio (UACR), and eGFR used in KFRE, eGFR was also used in FLEX-C, age and sex were removed due to weak feature expressiveness (‘Ablation experiments’ section in Supplementary Data), and UACR was replaced by the strongly correlated but more discriminatory urinary protein-to-creatinine ratio (UPCR) instead (‘Tabular datasets’ section in Supplementary Data). Thus, the clinical features used by FLEX-C are more representative and achieve higher performance.

During the selection of the optimal model architecture, we compared the effectiveness of different modal fusion methods in capturing inter-modal relationships (‘Ablation studies’ section in Supplementary Data). We evaluated four methods—summation, multiplication, gating, and concatenation—and obtained mean AUCs of 0.869, 0.562, 0.840, and 0.887, respectively (Fig. 2d). The lower mean AUC for the multiplication method is attributed to the fact that partial zero values in each modality’s representation vector result in ineffective information in the final multimodal representation. We also analyzed the performance of different image base models (‘Ablation studies’ section in Supplementary Data). Using ResNet18, ResNet34, ResNet50 and VGG-16 as base models, FLEX obtained mean AUCs of 0.887,0.866,0.876 and 0.873, respectively, suggesting that ResNet18 can extract image features better (Fig. 2e).

During data preprocessing, we also found that in clinical practice, missing data for one modality was common, so we developed the Meta module, which takes three other modalities as inputs and generates a replacement vector for the missing modality (‘Meta module’ section in Supplementary Data). Figure 2f shows that using the Meta module improves the average AUC of each modality by 1% compared to missing that modality. This indicated that FLEX is compatible with missing data and can be more effectively adapted for clinical applications.

### Impact of FET module on feature representation

To understand how the model enhances feature representation, we conducted ablation experiments focusing on the FET. We investigated the steps of the FET processing feature and reported the results in Table 1 (‘Ablation studies’ section in Supplementary Data). FET is a feature extractor capable of accepting data in tabular form as input from any data source. In this study, we applied FET to clinical data at individual level as well as proteomics data and metabolomics data at the molecular level. Firstly, we investigated the impact of ‘Hash mapping’. We found that processing data using ‘Hash mapping’ possesses a significant performance improvement compared to directly inputting the raw data (two-tailed Mann–Whitney U test P-value = 2.62 × 10^−5^). This finding validates our hypothesis that direct fusion of different levels of data is affected by noise due to distributional differences. Clinical data are mostly binomial and normal distributions, and proteomics and metabolomics data are mostly long-tailed distributions. ‘Hash mapping’ maps data of different modalities to the same scale and then aggregates the features, which partly preserves the original distribution and reduces the data complexity, thus improving the model generalization. Next, we investigated the impact of ‘Encoding training’. ‘Encoding training’ encodes the one-dimensional features generated by ‘Hash mapping’ into ten-dimensional vectors, and trains the feature encoding with gradient backpropagation and optimizer. The performance of ‘Encoding training’ similarly produced a significant improvement over the ‘Raw data’ (two-tailed Mann–Whitney U test P-value = 1.41 × 10^−5^). Specifically, ‘Encoding training’ increases the encoding length of the features to compensate the decrease in effective information due to ‘Hash mapping’. By train the encoding, with reference to the idea that the training model adjusts the parameters in order to extract effective data patterns, FET no longer fixes the feature encoding but implicitly learns the interrelationships of the features in the high-dimensional space through gradient computation. Each feature vector is additionally connected to a learnable fully connected layer to optimize feature representation more effectively. As shown in Table 1, the feature training approach not only enhances the model learning ability of the tabular data, but also captures the correlation between the image and the tabular data, realizing the end-to-end multi-modal fusion, thereby improving the performance of our model.

**Table 1:** Ablation experiment of FET

Modalities				Feature extraction		
C	P	M	I	Raw data	Hash mapping	Encoding training
√	√	√	√	0.818	0.856	0.887
	√	√	√	0.762	0.854	0.862
√		√	√	0.763	0.838	0.864
√	√		√	0.797	0.844	0.853
√	√	√		0.782	0.843	0.875
√	√			0.770	0.837	0.845
√		√		0.723	0.833	0.861
√			√	0.733	0.809	0.832
	√	√		0.716	0.857	0.863
	√		√	0.747	0.804	0.821
		√	√	0.769	0.830	0.833
√				0.671	0.800	0.805
	√			0.701	0.791	0.795
		√		0.794	0.828	0.831

Each row in the table has a check mark to indicate that the modality was used in the model and a space to indicate that the modality was removed. The evaluation metric is the mean AUC of the five-fold cross-validation.

### Interpretability and validation of multimodal prediction results

To further validate and interpret our model, we employed the feature aggregation and gradient-based methods to illustrate how each modal data affects the prediction (‘Model interpretability’ section in Supplementary Data). This section shows the reasonability of the features that the model focuses on by analyzing modal importance, image focus areas, and tabular feature importance.

**Figure 3:**
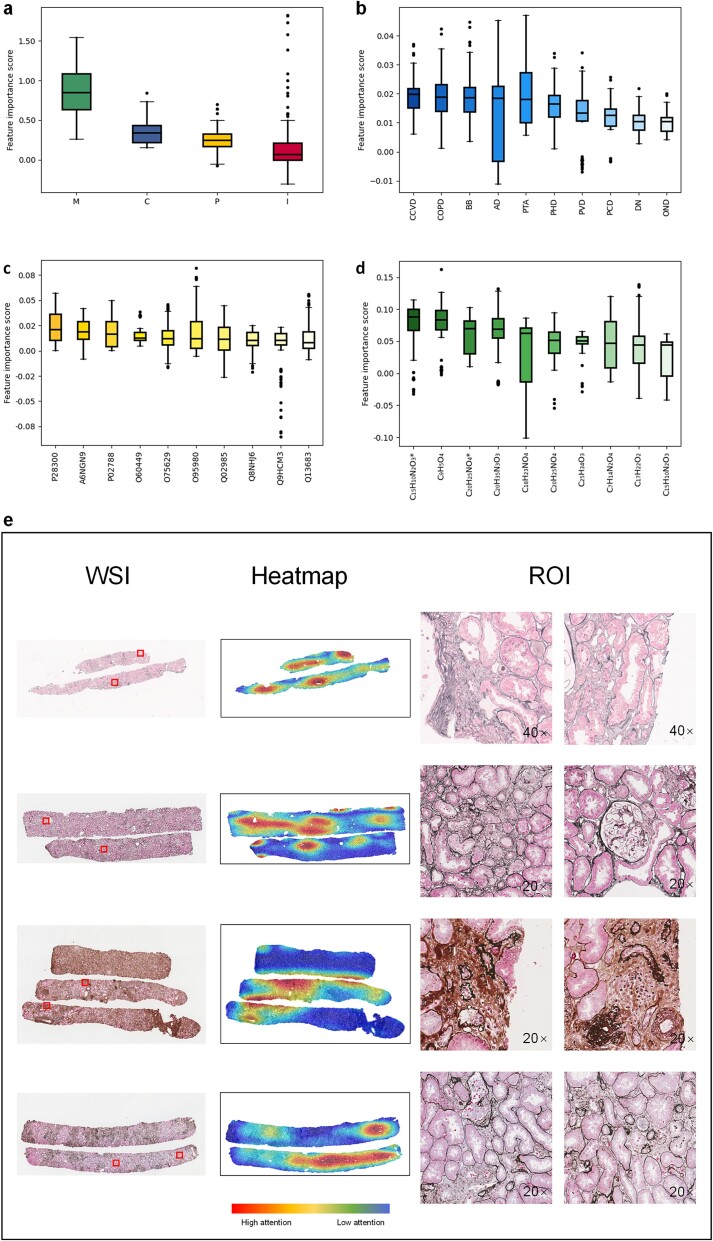
Interpretation of the output of the FLEX with progression group. (a) The modal importance of progression group (n = 106) is in descending order of metabolomics, clinical, proteomics, and images data. (b) Top 10 features in clinical data. (c) Top 10 features in proteomics data. (d) Top 10 features in metabolomics data (^*^ used to distinguish different metabolites with the same chemical formula). (e) Pathology images of whole slide images (WSI), heat map and regions of interest (ROI). The progression group was more focus on renal tubular atrophy, interstitial fibrosis, and white vacuoles in the tubules.

**Figure 4:**
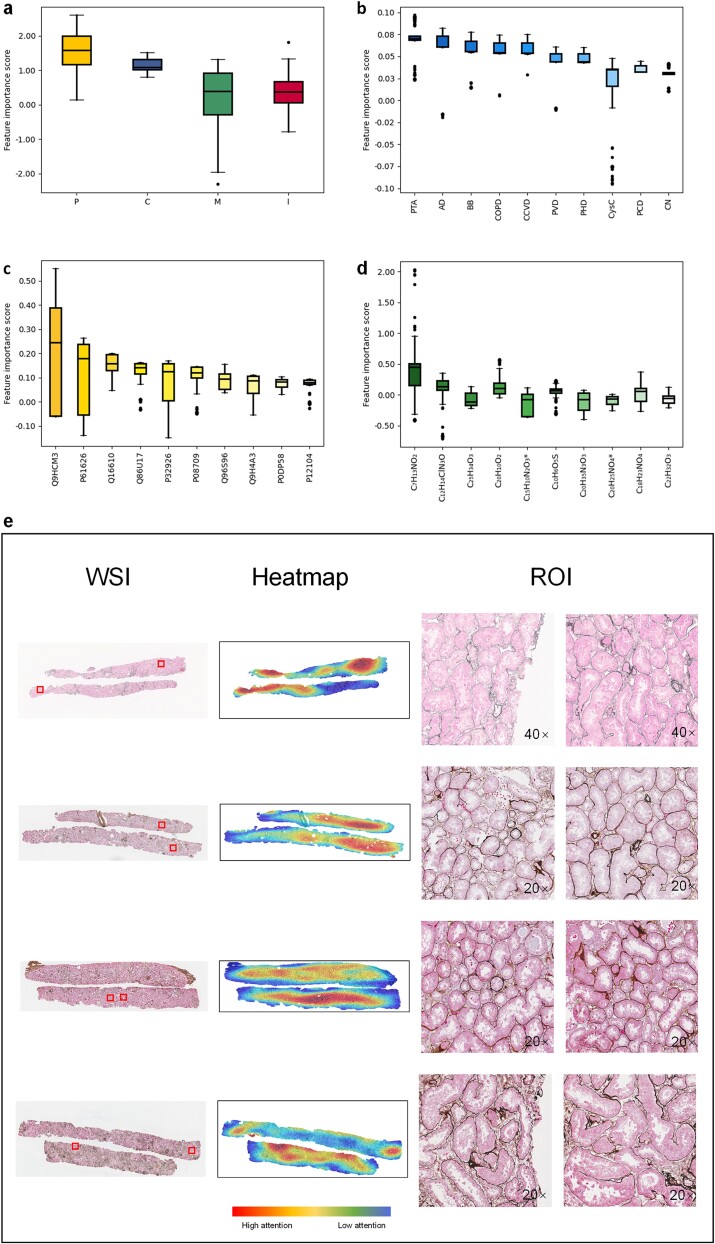
Interpretation of the output of the FLEX with non-progression group. (a) The modal importance of non-progression group (n = 153) is in descending order of proteomics, clinical, metabolomics, and images data. (b) Top 10 features in clinical data. (c) Top 10 features in proteomics data. (d) Top 10 features in metabolomics data. (^*^ used to distinguish different metabolites with the same chemical formula.) (e) Pathology images of whole slide images (WSI), heat map and regions of interest (ROI). The non-progression group was more focus on normal tissue areas.

**Figure 5:**
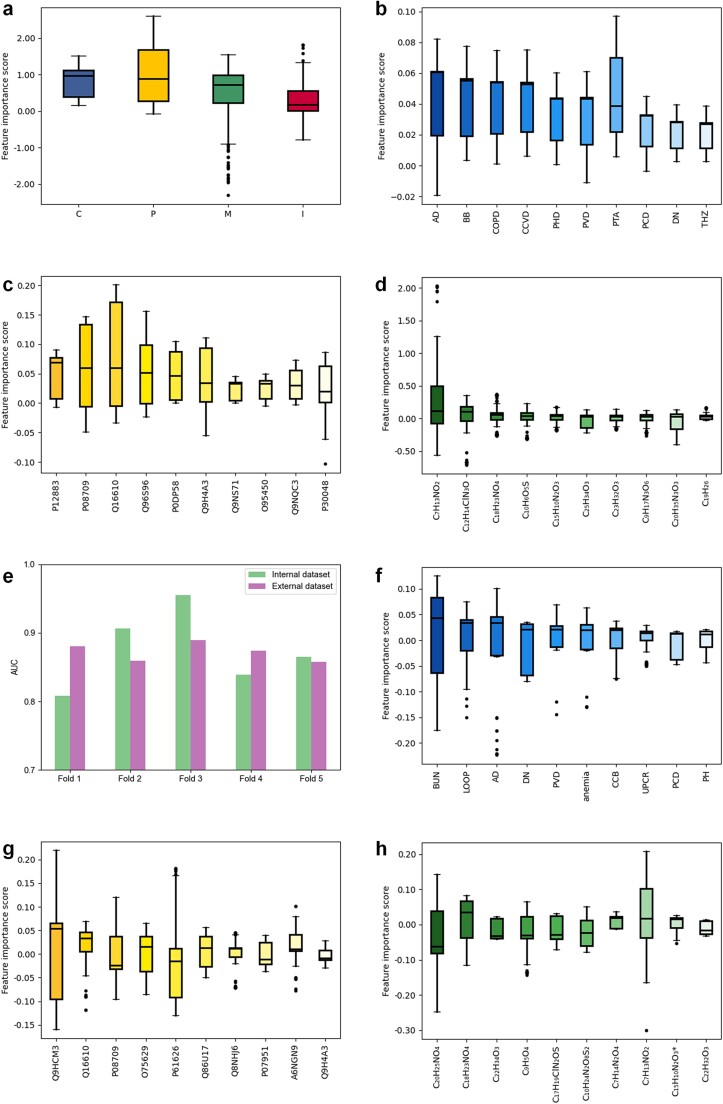
Validation of FLEX on internal and external datasets. (a) The modal importance of all patients is in descending order of clinical, metabolomics, proteomics, and images data on internal dataset. (b) Top 10 features in clinical data on internal dataset. (c) Top 10 features in proteomics data on internal dataset. (d) Top 10 features in metabolomics data on internal dataset. (e) Comparison of AUCs for tests in the internal and external datasets based on data from the three modalities. (f) Top 10 features in clinical data on external dataset. (g) Top 10 features in proteomics data on external dataset. (h) Top 10 features in metabolomics data on external dataset (^*^ used to distinguish different metabolites with the same chemical formula).

On the modal contribution evaluation, we used the gradient-based Integrated Gradients method [26]. This method calculates the impact of the representative features in each of the four modalities on the final prediction results. The degree of influence is reflected in the numerical values of the calculated results. We analyzed each modality for each patient and counted the distribution on different populations. For the internal dataset, we show the modal importance distributions for different populations in the progression group, non-progression group, and the whole internal dataset in Figs. 3a, 4a, and 5a, respectively. Notably, while the order of modal importance was not consistent for each population, the image data were ranked last, which suggests that we can reduce the use of kidney biopsy and then reduce the damage to the patient’s body.

Since the image modality had the lowest modal importance, we used the SCDA [27] to understand whether the image model learned effective morphological features. The SCDA method helps us to transform the model feature map into a score of the relevance of each pixel on the image to the outcome. We plot the heat map according to the score, with red representing strong correlation and blue representing weak correlation. The areas of high interest are selected and submitted to the doctors for validation and interpretability analysis. We show the whole-slide images (WSI), heatmaps, and regions of interest (ROI) in the images for the progression and non-progression groups in Figs. 3e and 4e, respectively. Interestingly, by comparing the regions of interest of the two groups, we found that the progression group focused more on regions of tubular atrophy and interstitial fibrosis, while the non-progression group focused on normal tissue regions, avoiding the severe lesions such as glomerulosclerosis. This implies that the image data also contributed effective and valuable information.

We additionally analyzed the importance of each indicator in the clinical data, proteomic data, and metabolomic data separately. Indicator importance analyses were also performed using the Integrated Gradients approach, where each indicator was treated as a separate feature input. We analyzed the importance of each feature for individual patients, thus comparing differences between features in different populations. Importance scores of features for all tabular data are in Supplementary Tables S3, S4, and S5. By ranking the features in descending order by the median importance score, we obtained the important features and validated them in the literature, thus enabling the interpretability analysis and result validation of the tabular data.

Figures 3b, 4b, and 5b highlight the top ten features in the clinical data. We observed that eight of the top ten features were present in all three populations, although they were in a different order. Five of the eight variables were labeled with a past medical history, including cardiovascular and cerebrovascular diseases (CCVD), chronic obstructive pulmonary diseases (COPD), pulmonary heart disease (PHD), peripheral vascular disease (PVD) and pulmonary circulatory disorders (PCD). Among these, cardiovascular comorbidities have been shown to be an important risk factor in increased risk of death for patients with CKD [28, 29]. COPD, known as a source of systemic inflammation and is similarly associated with the development of cardiovascular disease [30]. The involvement of cardiac, pulmonary, and vascular disease in the past medical history as key clinical features fits with the concept of managing comorbidities of cardiorenal and metabolic diseases in practice [31]. Additionally, the three remaining variables Beta-blocker medication history (BB) [32], antidiabetic drugs medication history (AD) [33], and prothrombin activity (PTA) [34] have all been studied in relation to their influence on the progression of CKD. In the complete list of ranked features, we also found that four features: past medical history of other neurological disorders (OND) [35], past medical history of primary hypertension (PH) [36], calcium-channel blocker medication history (CCB) [37], and Cystatin C (CysC) [38], showed significant variation in ranked position between the progression and non-progression groups. This suggest that these four features also show strong differentiation ability in predicting CKD progression. The complete character naming cross-reference table is in Supplementary Table S3.

Figures 3c, 4c, and 5c show the top ten ranked proteomic features. There is an overlap of important features in the progression group and in the whole internal dataset. For example, Q96S96, Q9H4A3, P08709, and Q16610 have been reported to be associated with IgAN [39], diabetic nephropathy [40], myocardial infarction in patients with CKD [41], and clear cell renal cell carcinoma [42], respectively. FLEX also identifies additional proteins associated with diabetic nephropathy (i.e. P12104 [43], O95980 [44], and P28300 [45]), or other types of kidney disease (i.e. P02788 [46], P61626 [47], and Q02985 [48]). Interestingly, this corresponds to the CKD pathologic diagnostic types that we involved in the construction of the internal dataset. We also found that two proteins O95450 [49] and P30048 [50] associated with renal cell carcinoma have high feature importance scores, which inspired us to migrate findings from other renal fields in CKD-related studies.

Figures 3d, 4d, and 5d demonstrate important metabolomic features. Comparing clinical and proteomic data, metabolomic features had the most distinctive gap between the progressive and non-progressive groups. Six of the top ten (C_15_H_10_N_2_O_3_*, C_9_H_5_O_4_, C_20_H_25_NO_4_*, C_20_H_35_N_3_O_3_, C_20_H_25_NO_4_, C_25_H_34_O_3_) in the progressive group were within the bottom eleven in the non-progressive group. Based on literature research, we believe that C_20_H_25_NO_4_* or C_20_H_25_NO_4_ stands in for Cilomilast, which ameliorates tubulointerstitial fibrosis [51]. There were also six of the top ten features in the non-progression group inside the bottom ten in the progression group (C_7_H_13_NO_2_, C_12_H_14_ClN_3_O, C_20_H_10_O_2_, C_10_H_6_O_5_S, C_19_H_26_, C_12_H_14_N_2_O_4_). C_7_H_13_NO_2_ be the proline betaine that reflects the osmoprotective role of the kidney [52]. C_18_H_23_NO_4_ is the only metabolite that shows significant importance across both populations. While there is less research on kidney metabolomics compared to proteomics and clinical data, our study indicates that metabolomic features are the most diverse. These findings could potentially lead to new insights and directions for kidney disease research.

### External validation

To further assess the generalization ability of the model, we validated the performance of FLEX on an external dataset. While the internal and external datasets had similar proportions of progression versus non-progression groups, there was a large difference in the characteristic’s statistics. The internal dataset had a relatively homogeneous distribution of eGFR, whereas the external dataset had 80.2% of patients with eGFR<60 ml/min/1.73m^2^ [53]. The most common pathological diagnostic category in the internal dataset was IgAN, and in the external dataset it was an unknown category in 69.1% of patients. We took the trained model on the internal dataset and tested it on the external dataset in order of five-fold cross-validation in turn. Both datasets in Fig. 5e demonstrate the best performance at the third fold, which may be due to the more reasonable feature distribution of the data. The mean AUCs for the internal and external datasets were 0.875 and 0.872, respectively, which were not significantly different.

We also validated important features of the clinical data (Fig. 5f), proteomics data (Fig. 5g), and metabolomics data (Fig. 5h), respectively. The clinical features of AD, DN, PVD and PCD performed consistently with the internal dataset. Among the remaining clinical characteristics, FLEX also focused on the biomarkers blood urea nitrogen [54] and UPCR [55], which assess kidney function. We still found associations between blood disorders and CKD in the external dataset, such as whether or not there was use of loop diuretics to control blood pressure [56], and whether or not there was a history of anemia [57] or PH [58]. CCB was the characteristic that differed significantly between the progression group and the non-progression group in the internal dataset. It ranked seventh in the external dataset, confirming our conclusion that it has strong differentiating ability. Proteomic variables Q16610, O95980, and P28300 have been discussed previously. P05451, A6NGN9, and O75629 are related to diabetic kidney disease [59], membranous nephropathy [60], and renal fibrosis [61], respectively. Several proteins, including Q9HCM3, Q86U17, Q8NHJ6, and O60449, have not been extensively studied yet and show considerable variation in their importance across different groups. We believe these proteins could open new avenues for research. In metabolomics, compounds such as C_7_H_13_NO_2_, C_18_H_23_NO_4_, and C_23_H_32_O_3_ exhibited consistent significance in our internal dataset. However, other features revealed the same trend observed in the internal dataset: population differences lead to significant variations in the distribution of feature importance.

## Discussion

The fusion computation of multimodal data in the medical field is an important research topic. Medical data come from a variety of individuals, measurement means, and application environments. Sources of data include but are not limited to clinical data, mobile health monitoring data, and demographic data. The difficulties caused by structural differences in data from different modalities lead to challenges in applying multi-modal algorithms to clinical practice. Traditional approaches divide the progression prediction problem into multiple components, but hinder learning of inter-modal relationships and make it difficult to optimize.

In this study, we present FLEX, a pioneering interpretable model designed for predicting CKD progression using multimodal data. As the first artificial intelligence model that integrates clinical, proteomic, metabolomic, and imaging data by enhancing feature representativeness, FLEX tackles the complex challenge of fusing data from diverse sources and scales. Its advanced architecture demonstrates exceptional performance in predicting CKD progression, highlighting its robustness and innovation. FLEX not only advances the state of the art in CKD prediction but also sets a new benchmark for handling multimodal data in clinical applications.

Our study shows that the prediction performance of multi-modal fusion exceeds that of unimodal prediction and the common clinical application methods. The performance tends to increase with the number of modalities. Particularly, the performance improvement from modal fusion is significant when there is large disparity in data feature extraction methods or referent information content between modalities. In the comparison experiments, the performance of FLEX is not significantly different under various groupings, demonstrating the model’s stability.

For tabular data, we have specifically developed the FET architecture. FET allows the model to learn feature encoding during modality fusion. Under the effect of gradient backpropagation, the feature encoding not only represents the information of individual features, but also contains the dependencies between the features. This feature training approach breaks the data format, model structure, and modal interaction constraints imposed by traditional early, intermediate, and late fusion approaches. Such dependencies and interactions are also reflected in the performance enhancement brought to the image modality by the fusion of FET with the image model. It is also indicated that FET can mine complementary semantic information thus assisting the image to find effective features. In addition, the ‘Hash mapping’ step of FET improves the model’s inclusiveness to different data distributions, resulting in application to a wider range of scenarios.

Images always play a less important role in clinical progress analysis, which is also reflected in our interpretability analysis. We validate the reasonableness of the model’s focus on features with known knowledge in feature importance analysis, and also provide new directions for the study of biomarkers.

In verifying the generalizability of FLEX, we compared the performance on an external dataset. The differences in dataset characteristics reflect clinical applications in real situations. Our model still demonstrates reliable capabilities.

Although we have validated the model from multiple perspectives, there are still some limitations to be aware of. From a clinical application perspective, we have developed the Meta module to cope with the problem of single modal missingness, but there are still cases of two or three modal missingness that have not yet been addressed. In terms of interpretability, although we have analyzed the distribution of feature importance within each modality, we do not know how a single feature can trigger a change in the model’s attention locations outside the modality.

In conclusion, this study validates that multimodal fusion is more effective than unimodal learning and underscores the importance of enhancing feature representativeness. FLEX, with its capabilities in feature learning and fusion, effectively complements its role in feature extraction, integration, and analysis of multimodal data. Looking ahead, expanding the application of these models to other diseases and engaging in collaborative validation studies will further demonstrate their versatility and effectiveness. These efforts will drive progress in early patient treatment planning and the timely allocation of medical resources, ultimately improving patient outcomes.

### Summary

In this paper, we present FLEX, an artificial intelligence multi-modal data fusion algorithm based on enhancing feature representativeness. FLEX can fuse clinical data, proteomic data, metabolomic data, and pathology images in the application scenarios of CKD progression prediction, and demonstrates superior performance. In addition, after comprehensive ablation experiments and interpretability analysis, FLEX demonstrates the stability and reasonableness of the prediction results. FLEX can be expanded to more data sources and application scenarios in the future, contributing to the promotion of the wide application of medical resources.

**Key Points**

- For the first time, we introduce the concept of enhancing feature representativeness into the development of medical multi-modal algorithms with FLEX. By aggregating features and optimizing encoding, FLEX effectively reduces distribution noise caused by modal heterogeneity while enriching feature information.
- During the algorithm experiments, we collected and organized clinical data, proteomic data, metabolomic data, and pathology images. FLEX is the first to use multi-source, multi-modal data on the problem of predicting chronic kidney disease progression.
- FLEX developed a new deep learning model structure for table-structured data called FET. FET allows for simultaneous training of the model structure and feature encoding, improving the model’s ability to capture interactions between different patterns and features in high-dimensional spaces.
- We validate the reliability of the model through detailed comparative experiments and feature importance analysis on internal and external datasets. The results showed that FLEX is robust, rational and generalizable.

## Supplementary Material

Supplementary_Data-proof_bbaf003

Supplementary_Tables-proof_bbaf003

## Data Availability

Availability of internal datasets and external datasets was restricted and permission to use these datasets in this study was obtained from the participants. The de-identified data can be requested from the corresponding author (xux007@163.com) for research purposes upon reasonable request. The code of this paper is available at: https://github.com/Qiaoyx97/FLEX.
