# Supplementary material for: A multi-modal fusion model with enhanced feature representation for chronic kidney disease progression prediction
Source: Brief Bioinform. 2025 Feb 6;26(1):bbaf003. doi: 10.1093/bib/bbaf003 (PMC11801269; doi:10.1093/bib/bbaf003)
Supplement: Supplementary_Data-proof_bbaf003 [file supplementary_data-proof_bbaf003.docx]

**Supplementary Data**

**Supplemental Figure**

**
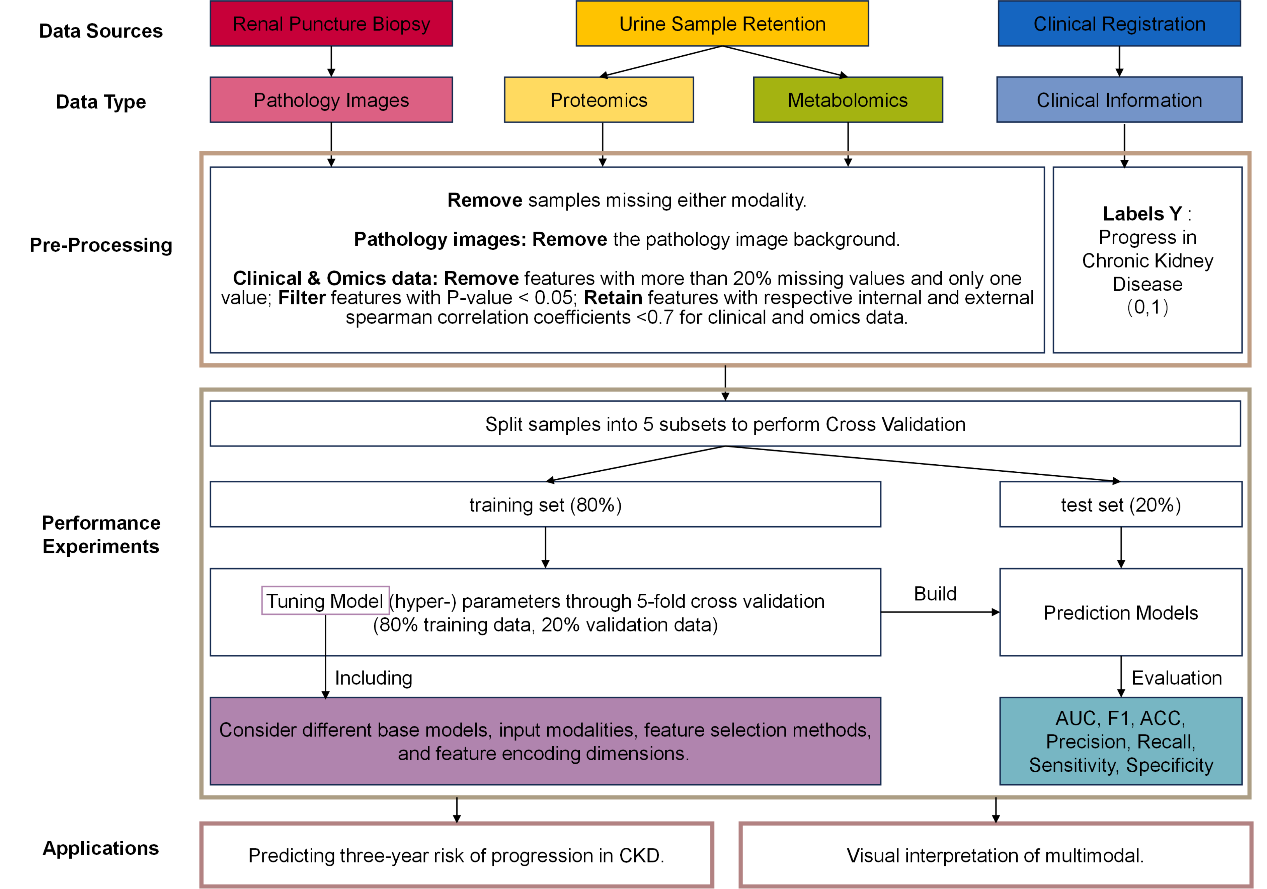
**

**Supplementary Figure S1 | Internal dataset processing flow diagram.**

**Methods**

**Data collection**

Our multi-modal datasets were all obtained from Nanfang Hospital, Southern Medical University. The Medical Ethics Committee of Nanfang Hospital, Southern Medical University approved the study protocol. All patients signed an informed consent form before kidney biopsy. All pathology images, electronic medical records, proteomics, and metabolomics data were stripped of personal information prior to algorithmic model development. Internal and external datasets were collected following the same standardized procedures, as detailed in Supplementary Table S8.

**Dataset description**

This study used an internal dataset involving 259 patients from Nanfang Hospital with chronic kidney disease. The patients' medical records started from May 2013 to November 2021 and had three-year follow-up records. Data were collected using the time of retention of the urine sample closest to the first creatinine test as the baseline time. Each sample contained three sources of data: kidney biopsy samples, electronic medical records, and urine samples. These samples and records generated data in four modalities: pathology images, clinical variables, proteomics data, and metabolomics data. We removed patients missing any of the modalities. The data processing flow is detailed in Supplementary Figure S1. The external dataset contained 81 patients at Nanfang Hospital from June 2013 through January 2022 and involved clinical data, proteomics data and metabolomics data.

**Progression definition**

All patients were categorized into progression and non-progression groups according to disease progression within three years based on follow-up records. Patients in the progression group were required to fulfill the following two criteria: (1) baseline eGFR (defined as the average creatinine within 30 days prior to the baseline time) greater than 25 mL/min/1.73 m²; (2) decline in eGFR of more than 40% with a progression of CKD stage by at least one level compared to before. Therefore, 106 of the 259 patients were in the progression group and defined as label 1. The remaining 153 patients were in the non-progression group and defined as label 0.

**Pathology images**

Each patient had a Periodic Acid-silver Methe-namine-stained image. In the image preprocessing stage, to avoid background impurities affecting the image quality, we used an automatic algorithm to segment the tissue and background regions. The image is converted from RGB to HSV color space and then Gaussian blur is applied to smooth the fine cavities. A binary mask is generated by binarizing the transparency channel of the image, and connected regions larger than 0.5% of the image area are saved as tissue regions. Following segmentation, all images are resized to 1024×1024.

**Tabular datasets**

Tabular datasets contain clinical, proteomics, and metabolomics data. The same data preprocessing method was used for all three modalities.

Tabular datasets were preprocessed in three parts. The first part was the selection of distinguishing variables within each modality. We first removed variables with more than 20% missing values or only one value. The remaining missing values were filled with the median of the variable. Variables whose data distribution changed before and after the filling of missing values were also removed. The remaining variables with a P-value of less than 0.05 for the two-tailed Mann-Whitney U test were considered statistically significant in distinguishing patients in the progression group from those in the non-progression group. These distinguishable variables consisted of 74 clinical variables, 2217 proteomic variables, and 3651 metabolomic variables. The second part was to remove features with strong covariates. We performed correlation analyses with Spearman's correlation coefficient within each modality and between clinical and omics data. If the absolute value of the correlation coefficient of any two variables was greater than 0.7, the variable with the smaller P-value was retained. The third part was using min-max normalization to scale continuous variables to between 0 and 1. After data preprocessing, we retained a total of 48 clinical variables, 1010 proteomics variables and 1617 metabolomics variables.

Proteomics data alignment was performed using the UniProt database [1] for protein-gene mapping. Metabolite chemical formula correspondence was accomplished through the HMDB database [2], selecting the chemical formula with the smallest delta based on m/z values as the representative for each metabolite.

**Model architecture**

For specific parameters, see Supplementary Table S9.

**Feature encoding trainer**

The Feature Encoding Trainer (FET) was utilized to extract and train features from clinical, proteomics, and metabolomics data presented in tabular format. Existing feature extractors can process either raw data input or encoded variables at the data preprocessing stage. We recognized that such a data processing approach would present significant challenges, particularly when dealing with multi-modal data with large scale gaps and a mixture of categorical and continuous variables. Directly inputting raw data may lead to issues with low generalizability, while preprocessing encoding methods, such as one-hot encoding, can result in several problems. There will be huge feature-encoding correspondence table, inability to accept features outside the correspondence table, and feature conflict problem (i.e., features with different meanings are encoded with the same encoding), and so on. Therefore, we proposed FET, which not only learns the abstract representation of each modality, but also learns the encoding of each feature.

FET first maps the raw variables $X=\left\{ x_{1},x_{2},\cdots x_{n} \right\}$ into $k$ categories $(k\leq10)$ by hash function $H\left( x \right)$:

$$H\left( x \right)=\left\{ \begin{aligned} x, &m\leq2 \\ \log_{2} \left( x+0.01 \right), &m>2 \end{aligned} \right.$$

where $m$ is the kinds of values contained in the original variable. This step, referred to as 'Hash Mapping,' operates similarly to binary or categorical feature encoding, but without introducing manual criteria. 'Hash Mapping' better preserves the original distribution information.

The mapped variables are then encoded as d-dimensional random vectors $\left\{ \mathbf{e}_{i} \right\}$, $\mathbf{e}_{i}\in\mathbb{R}^{d}$ (Supplementary Table S7). Next FET configures a fully connected layer for each vector $\mathbf{e}_{i}$ and allows the gradient to be back-propagated to the feature encoding layer, thus end-to-end updating and optimizing the feature encoding. The optimized feature encodings are concatenated for obtaining an entire encoding $\mathbf{E}$ of all features. Lastly two more fully connected layers followed by the ReLU activation are stacked for generating a 20-dimensional vector for representing the features of single modality. The generation of representative feature for clinical modality $\mathbf{C}_{p}\in\mathbb{R}^{20}$ can be expressed as:

$$\mathbf{E}_{p}=concat\left( W_{c,i,2}\mathbf{e}_{i,p}+\mathbf{b}_{c,i,2} \right), for i in [1,n]$$

$$\boldsymbol{C}_{p}=ReLU(W_{c,4}\left( \mathrm{ReLU}\left( W_{c,3}\mathbf{Z}_{p}+\mathbf{b}_{c,3} \right) \right)+\mathbf{b}_{c,4})$$

where $c$ means clinical modality, $W_{c,i,2}\in\mathbb{R}^{d\times d}$, $\mathbf{b}_{c,i,2}\in\mathbb{R}^{d}$, $W_{c,3}\in\mathbb{R}^{nd\times300}$, $\mathbf{b}_{c,3}\in\mathbb{R}^{nd}$ , $W_{c,4}\in\mathbb{R}^{300\times20}$ and $\mathbf{b}_{c,4}\in\mathbb{R}^{20}$. Proteomics representation vector $\boldsymbol{P}_{p}\in\mathbb{R}^{20}$ and metabolomics representation vector $\mathbf{M}_{p}\in\mathbb{R}^{20}$ are generated by the same process.

**Image feature extractor**

After completing the organizational segmentation step (see ‘Pathology images’) we chose ResNet18, pre-trained on ImageNet as the base of the image feature extractor to encode each 1024 × 1024 RGB image into a 1 × 512 low-dimensional feature embedding. During the encoding process, color normalized data augmentation was applied to the images. To facilitate subsequent multi-modal feature fusion, we used a fully connected layer, parameterized by $W_{1}\in\mathbb{R}^{512\times20}$ and $\mathbf{b}_{1}\in\mathbb{R}^{512}$, followed by the rectified linear unit (ReLU) activation to map an image embedding $\mathbf{Z}_{p}\in\mathbb{R}^{512}$ into a 20-dimensional vector $\mathbf{I}_{p}\in\mathbb{R}^{20}$ as a representation of the image modal feature for each patient *p*.

$$\mathbf{I}_{p}=ReLU(W_{1}\mathbf{Z}_{p}+\mathbf{b}_{1})$$

**Modality fusion and classification**

The representative vectors of each modality are first concatenated to fuse the multi-modal features. The concatenation results in an 80-dimensional feature vector that is fed into the classifier consisting of two fully connected layers to obtain patient-level prediction scores (softmax, softmax activation):

$$\boldsymbol{S}_{\boldsymbol{p}}=\mathrm{softmax}\left( W_{cls2}\left( \mathrm{ReLU}\left( W_{cls1}concat\left( \left[ \mathbf{I}_{p},\boldsymbol{C}_{p},\boldsymbol{P}_{p},\boldsymbol{M}_{p} \right] \right)+\mathbf{b}_{cls1} \right) \right)+\mathbf{b}_{cls2} \right)$$

where $W_{cls1}\in\mathbb{R}^{80\times20}$, $\mathbf{b}_{cls1}\in\mathbb{R}^{80}$, $W_{cls2}\in\mathbb{R}^{20\times2},\mathbf{b}_{cls1}\in\mathbb{R}^{20}$.

**Meta module**

FLEX requires data input in four modalities: image, clinical, proteomics, and metabolomics, but any of the modalities are often missing in clinical practice. Meta module is an optional plug-in module in FLEX to solve the incomplete data problem. When any modality is missing, the data from the remaining three modalities are used to generate replacement vectors for modality fusion. For example, if the image modality is missing, Meta takes clinical, proteomics, and metabolomics data as inputs and generates a 20-dimensional vector $\boldsymbol{R}_{p}$ through the full connectivity layer, which is then fused with the vectors $\boldsymbol{C}_{p},\boldsymbol{P}_{p},\boldsymbol{M}_{p}$ to obtain the patient-level predicted scores.

**Training details**

We perform five-fold cross-validation for all experiments, dividing 20% of the patients into the test set, and then dividing the remaining patients into the training and validation sets at a ratio of 8:2. The FLEX model was trained in an end-to-end fashion, with two Adam optimizers with a learning rate of 1×10^−3^ for the backbone network and FET, respectively. The backbone network’s Adam optimizer with weight decay of 1×10^−8^, and the remaining parameters for two optimizers were used with default settings. Additionally, the backbone network additionally utilized CosineAnnealingLR to smooth the learning rate variations. The experimental batch size is 8. During the training process, the performance of the model is measured with a cross-entropy function with weights of (1, 1.44). When training Meta module, weights summed two cross-entropy functions were used to optimize both the backbone network and the Meta module. Model performance on the validation set was monitored at each epoch, with early stopping employed if the validation loss did not improve for ten consecutive epochs. The model with the lowest validation set loss is regarded as the best model and its performance on the test set is reported. The classification performance is reported by the area under the receiver operating characteristic curve (AUC), precision, recall, accuracy (ACC), F1-score (F1), specificity. ROC curves were generated using the scikit-learn (v.0.24.2). The mean AUC of the experiment is the average of the five-test set AUCs. Model training and evaluation were performed with PyTorch (v.1.10.1) on a NVIDIA A100 GPU.

**Ablation studies**

We conducted ablation experiments to investigate the differences introduced by (1) feature numbers (Supplementary Table S6); (2) modal combinations (Supplementary Table S2); (3) modal fusion methods (Fig 2d); (4) base image models (Fig 2e) and (5) FET steps (Table 1). All models use the same hyperparameters and learning schedule as ‘Training details’ and are trained and tested using the same training: validation: test splits.

**Features numbers:** clinical, proteomics, and metabolomics variables, each ranked from smallest to largest according to the P-value of the two-tailed Mann-Whitney U test. The top 10, top 20, top 30, top 40, P-value<0.001, P-value<0.01, and P-value<0.05 features were sequentially selected as representative features of the modality, respectively, to compare the differences in performance from different numbers of features.

**Modal combinations**: Keeping the feature selection and model architecture unchanged, we selected three, two, and one modal data from the four modal data as input combinations to study the performance difference caused by different modal combinations.

**Modal fusion methods**: We used four methods of multimodal fusion including summation, multiplication, gating and concatenation methods to generate multimodal representations and compared the fusion performance.

**Base image models**: we compared the performance differences of using different base image models (VGG16 [3], ResNet18, ResNet34, ResNet50[4]) with constant input data.

**FET steps**: To verify the validity of FET, we analyzed the performance differences brought about by different steps, including hash mapping, encoding training, and vector encoding lengths (Supplementary Table S7), respectively, for various modal combinations on tabular datasets, while keeping the input features constant.

**Model interpretability**

**Feature Visualization of image**

To visualize the importance of each part of the image to the prediction results, we employed the SCDA [5] method to analyze the convolutional output from the last layer. We began by aggregating the convolution results across channels to create an attention score matrix. This matrix was then min-max normalized. Subsequently, the matrix was resized to match the original image dimensions, and a color map was applied to generate a heatmap. The heatmap was displayed with a transparency of 0.5, overlaid on the original image.

**Assessing modality contribution**

Each patient had four modal inputs, and we analyzed modal importance using the representative vectors ${\boldsymbol{I}_{p},\boldsymbol{C}}_{p},\boldsymbol{P}_{p},\boldsymbol{M}_{p}$ for each modality. Integrated Gradients (IG) [6] is a gradient-based feature importance analysis method that assigns an importance score to each representative vector. In A positive importance score represents a positive impact and a negative one represents a negative impact, and a larger absolute value of the score represents a larger impact on the prediction. Figures 2a, 3a, and 4a we show the distribution of importance of each modality in the progression group, non-progression group, and all patients, respectively, and the box plots are arranged in descending order by median.

**Evaluating tabular data features importance**

For tabular data, we evaluated feature importance by analyzing the impact of different features on model performance. Clinical, proteomic and metabolomic features were interpreted using the IG [6] method. The top ten features in each modality were ranked in descending order based on the absolute value of the median using a box plot.

**Reference**

1. UniProt, C., *UniProt: the Universal Protein Knowledgebase in 2023.* Nucleic Acids Res, 2023. **51**(D1): p. D523-D531.

2. Wishart, D.S., et al., *HMDB: the Human Metabolome Database.* Nucleic Acids Res, 2007. **35**(Database issue): p. D521-6.

3. Simonyan, K. and A. Zisserman, *Very Deep Convolutional Networks for Large-Scale Image Recognition.* CoRR, 2014. **abs/1409.1556**.

4. He Kaiming, Z.X., Ren Shaoqing, Sun Jian, *Deep Residual Learning for Image Recognition.* 2016 IEEE Conference on Computer Vision and Pattern Recognition (CVPR), 2016: p. 770-778.

5. Wei, X.-S., et al., *Selective Convolutional Descriptor Aggregation for Fine-Grained Image Retrieval.* Trans. Img. Proc., 2017. **26**(6): p. 2868–2881.

6. Sundararajan, M., A. Taly, and Q. Yan, *Axiomatic attribution for deep networks*, in *Proceedings of the 34th International Conference on Machine Learning - Volume 70*. 2017, JMLR.org: Sydney, NSW, Australia. p. 3319–3328.
